# Structural Insights into Calcium-Bound S100P and the V Domain of the RAGE Complex

**DOI:** 10.1371/journal.pone.0103947

**Published:** 2014-08-01

**Authors:** Srinivasa R. Penumutchu, Ruey-Hwang Chou, Chin Yu

**Affiliations:** 1 Department of Chemistry, National Tsing Hua University, Hsinchu, Taiwan; 2 Graduate Institute of Cancer Biology and Center for Molecular Medicine, China Medical University, Taichung, Taiwan; 3 Department of Biotechnology, Asia University, Taichung, Taiwan; 4 The Key Laboratory for Chemical Biology of Fujian Province, College of Chemistry and Chemical Engineering, Xiamen University, Xiamen, China; National Research Council of Italy, Italy

## Abstract

The S100P protein is a member of the S100 family of calcium-binding proteins and possesses both intracellular and extracellular functions. Extracellular S100P binds to the cell surface receptor for advanced glycation end products (RAGE) and activates its downstream signaling cascade to meditate tumor growth, drug resistance and metastasis. Preventing the formation of this S100P-RAGE complex is an effective strategy to treat various disease conditions. Despite its importance, the detailed structural characterization of the S100P-RAGE complex has not yet been reported. In this study, we report that S100P preferentially binds to the V domain of RAGE. Furthermore, we characterized the interactions between the RAGE V domain and Ca^2+^-bound S100P using various biophysical techniques, including isothermal titration calorimetry (ITC), fluorescence spectroscopy, multidimensional NMR spectroscopy, functional assays and site-directed mutagenesis. The entropy-driven binding between the V domain of RAGE and Ca^+2^-bound S100P was found to lie in the micromolar range (K_d_ of ∼6 µM). NMR data-driven HADDOCK modeling revealed the putative sites that interact to yield a proposed heterotetrameric model of the S100P-RAGE V domain complex. Our study on the spatial structural information of the proposed protein-protein complex has pharmaceutical relevance and will significantly contribute toward drug development for the prevention of RAGE-related multifarious diseases.

## Introduction

The receptor for advanced glycation end products (RAGE) is a cell surface signaling receptor and a member of the immunoglobulin superfamily [1], [2]. RAGE is composed of an N-terminal variable-type (V) domain, two distinct C-type Ig-like domains (C1 and C2), a transmembrane helix domain (TMH) and a highly charged cytoplasmic tail [3]. The V-type domain is generally involved in ligand binding, and the highly charged cytoplasmic tail is associated with the activation of intracellular signal transduction pathways [4]. This signaling receptor is involved in a wide range of inflammation-related pathological states, such as vascular diseases, diabetes, neurodegeneration and cancer [5], [6], [7], [8]. The activation of RAGE and the signal transduction that follows is also dependent on the cell type and ligand concentration [9], [10]. Understanding RAGE signaling is invaluable for the prevention of various diseases. RAGE can interact with a variety of ligands, including advanced glycation end products (AGE) [1], [2], DNA [11], amphoterin (HMGB1) [12], β-amyloid [13] and S100 family proteins [14], [15]. RAGE ligation and its subsequent activation play a role in multiple signaling cascades, such as the MAPK, JNK and Cdc42/Rac pathways, and activate the transcription factors AP-1 and NF-κB [16], [17], [18]. Previous studies have suggested the possibility of RAGE TMH dimerization during signal transduction [19], [20], [21]. The homodimerization of RAGE is an important step for receptor activation during ligand binding and, thus, for the induction of various signaling cascades [22], [23]. The ligation of RAGE by its targets, such as S100B and AGEs, leads to the enhanced formation of RAGE homodimers and is also associated with amplified signal transduction and transcriptional activation [24].

S100P is a member of the S100 family of small calcium-binding proteins and has been reported to possess both intracellular and extracellular functions [25], [26]. S100P binds to the extracellular region of RAGE and activates various signaling pathways, including the downstream pathways of mitogen-activated protein kinase (MAPK), serine protein kinase (SK), extracellular signal-regulated kinase (ERK) and nuclear factor-kappa B (NF-κB) [9], [27], [28]. The ligation of RAGE by S100P leads to cell proliferation and survival to mediate tumor development [29]. The physiological interaction between S100P and RAGE has been demonstrated by co-immunoprecipitation in different cell types, including embryonic fibroblast [28], pancreatic cancer cells [30], [31], and colon cancer cells [29]. Suppression of RAGE by different methods, such as dominant negative mutant of RAGE (DnRAGE), anti-RAGE antibody, and RAGE antagonist peptide, effectively inhibited S100P-induced cell proliferation, indicating that S100P signals mostly through RAGE [28]. Previous studies revealed that amphoterin-derived peptides and S100P-derived antagonist peptides are known to block the interactions between S100P and the V domain of RAGE [29], [32]. Recently, the anti-allergic drug cromolyn sodium and its analogs have also been shown to block the interactions between RAGE and S100P [33], [34]. The conformational changes of S100P that occur upon binding to Ca^2+^, Mg^2+^ and Zn^2+^ have been characterized using circular dichroism (CD), fluorescence spectroscopy, size exclusion chromatography and equilibrium analytical ultracentrifugation [35]. Calcium-bound S100P has been shown to bind various peptides, such as mellitin and TRTK12 [36], [37], [38]. In the intracellular space, the S100P homodimer binds and activates the cytoskeletal proteins ezrin and IQGAP1 [26], [39]. The interaction between S100P and CacyBP/SIP has also been reported to lead to β-catenin degradation [40].

S100 proteins are known to bind to diverse targets and utilize the hydrophobic patch present on the S100 linker region (between helices 2 and 3) and either helix-4 or the C-terminus. However, the sequence variability in the linker region between helix-2 and helix-3, helix-4, and the extended C-terminal region of S100 proteins with an altered net charge and polarity of their binding interface allows for the interaction with a wide range of target proteins [41], [42]. As observed for other S100 proteins, S100P utilizes the linker region and/or the extended C-terminal region to recognize specific target binding partners, such as mellitin [36], the TRTK12 peptide [38] and ezrin [26]. The deletion of hydrophobic residues, such as Y88 and F89, at the C-terminus of S100P abolishes its binding to the cytoskeletal protein ezrin. In addition, S100P is also known to bind to IQGAP1 with its first EF-hand loop region constituting a portion of the binding site [39]. The difference in the binding mode and flexibility of S100P differentiates the mechanism of S100P recognition of the RAGE V domain from the recognition of interaction partners by other S100 proteins. Therefore, we sought to map the structural features of the complex of S100P and the V domain of RAGE. These features will provide important insights into the roles of specific S100P residues in defining the distinct binding mode of this protein with the V domain of RAGE and other known protein targets.

In this study, we demonstrate that two RAGE V domains bind to two symmetrical interfaces in the calcium-bound S100P homodimer, forming a heterotetrameric complex. This complex further leads to RAGE homodimerization and activation of downstream signaling pathways, such as ERK, NF-κB, and MAPK pathways. The binding affinity for the interaction between S100P and the V domain of RAGE was determined by fluorescence spectroscopy and isothermal titration calorimetry (ITC). A HADDOCK approach was used with the interaction sites as constraints to model the structure of the S100P-RAGE V domain complex using a previously determined X-ray crystallographic structure of calcium-bound S100P [43] and an NMR solution structure of the V domain of RAGE [44]. Furthermore, to understand the role of the binding interface residues of S100P in its interactions with the V domain of RAGE, we performed a mutagenesis study on wild-type S100P and characterized the mutant S100P proteins using ITC and mitogenic assays. We also identified pentamidine as a small molecule that can bind to S100P and inhibit the interactions between S100P and the RAGE V domain, according to our HADDOCK binding model. Finally, we elucidated the structure of the binding interface of the S100P-RAGE V domain complex. These results offer a new perspective to our understanding of the binding mode of calcium-bound S100P to RAGE and reveal the mechanism of target binding partner recognition. The calculated model of the S100P-RAGE V domain complex provides important insights for the design of improved drugs to prevent several diseases.

## Materials and Methods

### 2.1 Reagents and Chemicals

The reagents for Luria broth were obtained from AMRESCO. ^15^NH_4_Cl, ^13^C-labeled glucose, and D_2_O were purchased from Cambridge Isotope Laboratories, and β-mercaptoethanol was obtained from Sigma. SW-480 cells were purchased from the American Type Culture Collection (ATCC; CCL-228).

### 2.2 Expression and Purification of S100P and the V domain of RAGE

Recombinant wild-type S100P (residues 1–95), the single mutants S100P E5A and S100P D13A and the triple mutant S100P F44G/Y89G/F89G were cloned into the pET-20b(+) expression vector, overexpressed in BL21(DE3) host cells and purified as previously reported [45]. All S100P proteins eluted as a dimer (in the final fractions) from size exclusion chromatography using a Superdex 75 column (1.6×60 cm; Pharmacia) and were concentrated in 20 mM Tris-HCl (pH 7.0), 100 mM KCl and 4 mM CaCl_2_. The cDNA encoding the recombinant RAGE V domain (residues 24–121) was subcloned into the pET-15b(+) expression vector, transformed into BL21(DE3) Codon Plus host cells and expressed and purified as previously described [3]. Briefly, following cobalt affinity chromatography, the 6-histidine tag at the N-terminus of the RAGE V domain was cleaved with thrombin at 25°C for 3 h. The cleaved RAGE V domain was further purified as a monomer by size exclusion chromatography using a Superdex 75 column (1.6×60 cm; Pharmacia) that was equilibrated with 20 mM Tris-HCl (pH 7.5), 100 mM KCl and 4 mM CaCl_2_. The eluted fractions were concentrated using Ultra-Centricon filters (Millipore) to a protein concentration of 0.7–1.0 mM. The purity was estimated to be approximately 95% by Coomassie-stained SDS-PAGE and HPLC analyses. The molecular weight was confirmed by ESI-TOF mass spectrometry.

### 2.3 NMR chemical shift assignments

All NMR experiments were performed at 298 K on a Varian 700 MHz spectrometer equipped with a cryogenic triple-resonance probe. The backbone and side chain chemical shifts of calcium-bound S100P have been previously assigned and have been deposited in the BMRB [45]. The backbone and side chain resonance assignments for the RAGE V domain have been previously reported under different buffer conditions. However, the backbone and side chain assignments were further examined in 20 mM Tris-HCl (pH 7.0), 100 mM KCl and 4 mM CaCl_2_ using ^1^H-^15^N HSQC, ^1^H-^13^C HSQC, HNCA [46], HN(CO)CA [47], CBCA(CO)NH [48], HNCACB [49], HNCO [50], HBHA(CO)NH [51], ^15^N-edited TOCSY [52], HCCH–COSY and HCCH-TOCSY [53]. The data were processed using VnmrJ 2.3 software and analyzed using Sparky 3.1 [54].

### 2.4 Isothermal Titration Calorimetry

For the preparation of protein samples, respective samples of S100P and the V domain of RAGE were dialyzed in 20 mM Tris-HCl (pH 7.0), 100 mM NaCl and 4 mM CaCl_2_ for 36 h with three buffer exchanges using a dialysis membrane with a molecular weight cut-off of approximately 3500 Da. All proteins samples were centrifuged and degassed under vacuum for 15 min to remove air bubbles prior to the titration experiment. ITC experiments were performed using a MicroCal VP-ITC calorimeter. A 2.0 mM solution of S100P was placed in a syringe as the protein ligand and titrated into a sample cell containing a 0.06 mM solution of the RAGE V domain. All titrations were performed at 25°C, and an injection volume of 10 µL was used for all subsequent titrations, with a 60-s initial equilibration delay and a 280 s delay in the intervals between the injections. The titration curves were corrected using buffer controls and analyzed using Origin software (MicroCal). A similar approach was used for subsequent experiments to characterize the interactions between mutant S100P proteins and the V domain of RAGE.

### 2.5 Fluorescence experiments

An emission spectrum for the intrinsic tryptophan fluorescence of the RAGE V domain was obtained using a Hitachi F-2500 fluorescence spectrophotometer. The tryptophan in the RAGE V domain was excited at a wavelength of 295 nm, and subsequent changes in the emission spectra were monitored by scanning from 305 to 400 nm with a slit width of 5 nm. A solution of S100P was added in 2-µL increments to a 2 µM solution of the RAGE V domain in 20 mM Tris-HCl (pH 7.0), 100 mM NaCl, and 4 mM CaCl_2_ at 25°C. After each addition of S100P, the sample was stirred for 2 min prior to scanning. The changes in the fluorescence intensities (ΔF) were monitored at 348.5 nm. S100P does not contain tryptophan residues, and the tyrosine and phenylalanine residues in S100P do not interfere with the excitation wavelength used here. The ΔF versus [S100P] plots were then fitted using the Origin program with equation 1 [55] as follows:
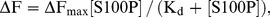
(1)where K_d_ is the dissociation constant and ΔF_max_ is the maximal fluorescence change.

### 2.6 NMR Experiments

The NMR HSQC titrations were monitored by recording 2D ^1^H-^15^N HSQC spectra at 25°C on a Varian 700 MHz spectrometer equipped with a cryoprobe. For HSQC characterization, all protein samples were prepared at a concentration of 0.3 mM in 20 mM Tris-HCl (pH 7.0, 10% D_2_O) containing 100 mM KCl and 4 mM CaCl_2_. Unlabeled calcium-loaded S100P was added to uniformly ^15^N-labeled RAGE V domain at molar ratios of 1∶0.2, 1∶0.4, 1∶0.6, 1∶0.8, 1∶1, 1∶2, 1∶3 and 1∶4. Similar mixtures of unlabeled RAGE V domain and uniformly^ 15^N-labeled S100P were also prepared. The shift and/or missing peaks were identified by overlaying the HSQC spectra. The observed changes in the cross-peak intensities of the ^15^N-labeled S100P-RAGE V domain complex were compared to free S100P and plotted in a bar graph. Furthermore, 3D ^13^C(ω2)-edited, ^12^C(ω3)-filtered NOESY-HSQC spectra were acquired with a mixing time of 120 ms using doubly labeled (^15^N, ^13^C) RAGE V domain mixed with unlabeled (^14^N, ^12^C) S100P at a 1∶1 molar ratio [56]. The concentration of each protein sample was 0.6 mM in a solution containing 20 mM Tris-HCl (pH 7.0), 100 mM KCl and 4 mM CaCl_2_. The final sample was lyophilized and then dissolved in >99% D_2_O.

### 2.7 Docking Calculations

The model of the S100P-RAGE V domain complex was generated using the HADDOCK2.0 web server with CNS 1.1 [57]. The structural coordinates for calcium-bound S100P and the V domain of RAGE were obtained from the RCSB Protein Data Bank (PDB), entries 1J55 and 2E5E, respectively. The decrease in the cross-peak intensities from ^15^N-^1^H HSQC analysis observed upon complex formation were used to define the ambiguous interaction constraints for residues at the interface. Active residues were defined as residues that exhibited the maximum decrease in the intensity of a cross-peak and a relative residue accessible surface area larger than 30% for either side chain or backbone atoms, as calculated by NACCESS [58]. Passive residues were defined as all other surface non-accessible residues exhibiting a relative residue accessible surface area of less than 30% for side chain or backbone atoms. Intermolecular NOEs are the most reliable source to define the intermolecular protein-protein complex structural information. Ten intermolecular NOEs were assigned from 3D ^13^C(ω2)-edited, ^12^C(ω3)-filtered NOESY-HSQC analysis, and these structural data were used as input for the HADDOCK calculations. That is, unambiguous restraints derived from 10 assigned NOEs were defined as intermolecular constraints between S100P and the V domain of RAGE during the docking calculations. A total of 2000 rigid-body docking trials were performed using the standard HADDOCK protocol with optimized potentials for liquid simulations (OPLS) parameters [59]. In addition, NCS (non-crystallographic symmetry) constraints were used for the symmetrical heterotetrameric complex calculations to enforce between the S100P and RAGE V domain molecules and the C2 symmetry restraints defined within the S100P homodimer. The 200 lowest energy solutions were used for subsequent semi-flexible simulated annealing and additional explicit water refinement. The 10 lowest energy structures were used to represent the structure of the complex according to the average interaction energy and buried surface area and were analyzed using PROCHECK [60]. Visualization and structural representations were rendered using PyMOL (DeLano Scientific LLC) [61].

### 2.8 Cell Culture

The human colorectal adenocarcinoma cell line SW-480 (ATCC: CCL-228) was cultured in DMEM/F12 supplemented with 10% fetal bovine serum, 100 U/ml penicillin, and 100 µg/ml streptomycin in a humidified incubator with 5% CO_2_ at 37°C.

### 2.9 Cell Proliferation Assay

Cell proliferation was measured using an MTT (3-[4,5-dimethylthiazol-2-yl]-2,5-diphenyltetrazolium bromide) assay. The day before the experiment, SW-480 cells were seeded at a density of 1×10^4^ cells/well in a 96-well cell culture plate. Subsequently, the cells were treated with 100 nM protein (wild-type S100P, S100P E5A, S100P D13A or S100P F44G/Y88G/F89G) and 10 µM FPS-ZM1 for 48 h. MTT solution was then added to each well to a final concentration of 0.5 mg/ml, and cells were incubated at 37°C for an additional 3 h. After removal of the medium, formazan crystals were dissolved in 100 µL of DMSO (dimethyl sulfoxide) by gentle agitation on a shaker for 10 min. The absorbance was measured at 570 nm using a Synergy 2 microplate reader (BioTek Instruments, Inc., VT, USA). The cell counts were determined from the relative absorbance of the experimental treatment compared with the control treatment.

## Results

### 3.1 Isothermal titration calorimetry

**Figure 1:**
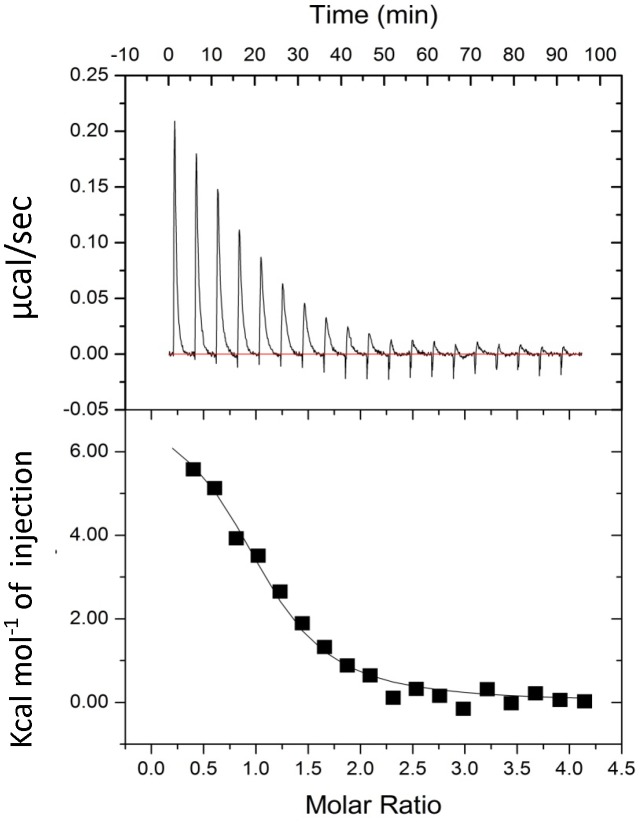
ITC titrations of the RAGE V domain with S100P. The raw thermogram and binding isotherm of S100P binding to the RAGE V domain at 25°C. The upper panel represents the raw data and whereas the bottom panel is the integrated plot of the amount of heat liberated per injection as a function of the molar ratio of the WS100P to RAGE V Domain. The concentrations of the RAGE V domain and S100P used in the ITC experiments were 0.06 mM and 2.0 mM, respectively. The changes in the heat during each injection of S100P into a solution of the RAGE V domain fit a single-site binding model with a K_d_ of approximately 6 µM. The titrations were performed in 20 mM Tris-HCl (pH 7.0) containing 4 mM CaCl_2_.

Isothermal titration calorimetry (ITC) experiments were performed to assess the binding affinity and stoichiometry of the complex by determining the heat changes that occur during protein-protein binding [62], [63]. The binding isotherm for the interaction between S100P and the V domain of RAGE is presented in **Figure 1**. The data were fitted by a nonlinear least squares approach to the ‘one set of sets’ site model and yielded a dissociation constant of K_d_ = 6.2±0.1 µM with additional parameters of N = 1.07±0.03, ΔH = 4.094±0.02 kcal/mol, and ΔS = 37.6 cal/mol/deg. The 1∶1 stoichiometry of the binding interaction is evident from the existence of two identical binding sites between two V domains of RAGE and the S100P homodimer. Additionally, the formation of a symmetrical heterotetrameric S100P-RAGE V domain complex was inferred from the single-binding site model, as evident from the ITC binding isotherm. The thermodynamic factors ascertained for the interaction of S100P with the RAGE V domain signified that their binding is stabilized by entropic factors and counter affected by a positive change in the enthalpy of binding. The positive change in entropy, ΔS (favorable), and positive change in the heat capacity of the system, ΔH (unfavorable), suggested that the binding was driven by entropy and supports the role of the hydrophobic residues present at the interface between the RAGE V domain and S100P in complex formation. The favorable entropic contribution to the stability of the S100P-RAGE V domain complex is likely due to the entropy gain from the burial of the hydrophobic patches at the interface and the displacement of water from the nonpolar surfaces of both S100P and the V domain of RAGE. Overall, our findings from ITC analysis of the interaction between S100P and the V domain of RAGE are consistent with previous studies [64], [65].

### 3.2 Intrinsic tryptophan fluorescence measurements

**Figure 2:**
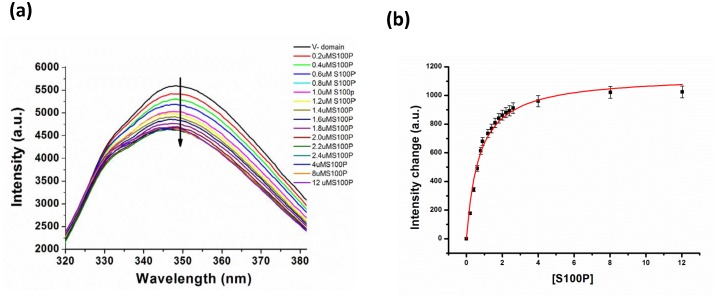
Interactions of the RAGE V domain with S100P monitored using fluorescence spectroscopy. (a) Fluorescence emission spectra of the RAGE V domain illustrating the changes in the intrinsic tryptophan fluorescence with an increasing concentration of S100P in micromolar range. (b) Changes in the fluorescence intensities measured at 348.5 nm as a function of the S100P concentration was calculated according to Eq. 1. The binding was fit (solid red line) to a one-site binding model.

The intrinsic tryptophan fluorescence of proteins is sensitive to the polarity of the local environment and conformational changes in the protein associated with substrate binding and can be monitored [66]. Tryptophan residues exposed to highly polar environments exhibit emission maxima in the range of 345 to 360 nm upon excitation at a wavelength of 295 nm, whereas tryptophan residues in hydrophobic environments exhibit emission maxima ranging from 330–345 nm [67]. The V domain of RAGE contains three tryptophan residues at positions 51, 61 and 72. An analysis of the relative solvent accessibility of these three tryptophan residues indicated that W51 and W72 are buried inside the hydrophobic core of the RAGE V domain. However, W61, which is exposed to the solvent and is present at the binding interface of the S100P-RAGE V domain complex, can serve as a suitable probe for the fluorescence characterization of this interaction. Furthermore, the RAGE V domain exhibits an emission maximum at 348.5 nm upon excitation at 295 nm [68]. Thus, the change in the tryptophan fluorescence intensity of the RAGE V domain at 348.5 nm was monitored upon excitation at 295 nm. Titration of the RAGE V domain with S100P resulted in a decrease in the intrinsic tryptophan fluorescence of the RAGE V domain, as shown in **Figure 2a**. Fitting of the fluorescence intensity change versus the S100P concentration to a one-site binding model, as shown in **Figure 2b**, yielded a dissociation constant, K_d_, of approximately 6.8 µM. Overall, our fluorescence results are consistent with our ITC analysis and suggest a modestly strong binding affinity of S100P for the RAGE V domain that is in the lower micromolar range, confirming the formation and stability of this protein complex under physiological conditions.

### 3.3 Mapping the binding interface between S100P and the V Domain of RAGE

**Figure 3:**
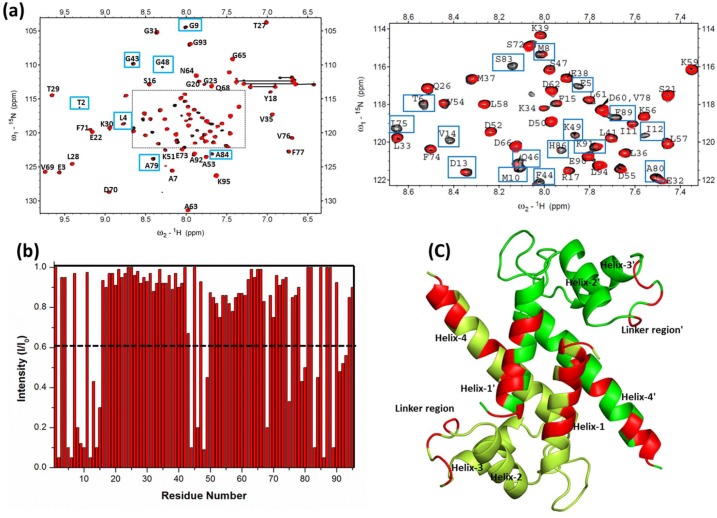
Analysis of the ^1^H-^15^N HSQC spectra of S100P in complex with the V domain of RAGE. (a) Overlaid ^1^H-^15^N HSQC spectra of 0.3 mM ^15^N-labeled free S100P (black) and S100P in complex with 0.3 mM unlabeled RAGE V domain (red), with spectral changes indicated by blue boxes. The dashed line indicates the expansion spectra shown on the right. (b) Bar graph representing the changes in the cross-peak intensities (I/I_o_) of free S100P and S100P in complex with the RAGE V domain versus the S100P residue number (1-95). In this plot, (I) represents the cross-peak intensity of S100P in complex with the RAGE V domain and (I_o_) represents the initial intensity of free S100P. The black dashed line indicates the threshold of the selected residues that exhibited a significant decrease in the intensity (<0.6). (c) Ribbon representation of the S100P homodimer with the residues that exhibited a decrease in the cross-peak intensity was mapped (red color) in the ribbon diagram. The monomers of the S100P homodimer are colored green and lemon green.

**Figure 4:**
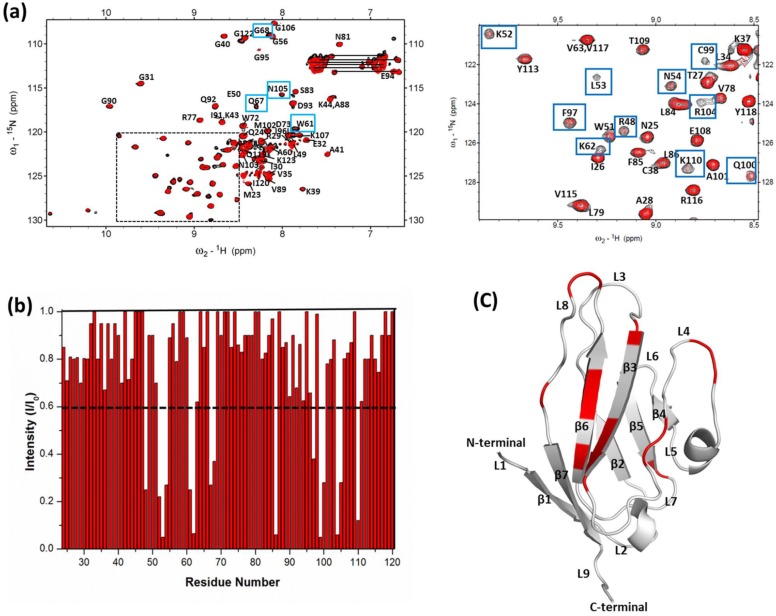
Analysis of the ^1^H-^15^N HSQC spectra of the RAGE V domain in complex with S100P. (a) Overlaid ^1^H-^15^N HSQC spectra of 0.3 mM free ^15^N-labeled RAGE V domain (black) and the RAGE V domain in complex with 0.3 mM unlabeled S100P domain (red). Spectral changes are indicated by blue boxes. The dashed line indicates the expansion spectra shown on the right. (b) Bar graph of the cross-peak intensity (I/I_o_), where (I) is the cross-peak intensity of the RAGE V domain in complex with S100P and (I_o_) is the initial intensity of the free RAGE V domain, versus the RAGE V domain residue number (residues 21-121). The residues are numbered as previously reported [44]. The black dashed line is the threshold of the selected residues that exhibited a significant decrease in intensity (<0.6). (c) Ribbon representation of the RAGE V domain, with the residues that exhibited a decrease in the cross-peak intensity was mapped (red color) in the ribbon diagram. β-strands and loops are labeled in black.

Two-dimensional ^1^H-^15^N HSQC NMR is a useful technique to identify the protein-protein or protein-ligand binding interface [69]. This technique provides valuable information to characterize the exchange regime of binding [70], [71], [72]. Residues at the interface between calcium-bound S100P and the V domain of RAGE were identified by observing the changes in the line shape and width of the resonances in the ^1^H-^15^N HSQC spectra of free S100P compared with S100P in complex with the RAGE V domain. Overlaid HSQC spectra of ^15^N-labeled free S100P and S100P in complex with the unlabeled RAGE V domain are shown in **Figure 3a**. Differential line broadening of the NMR signals for S100P in complex with the RAGE V domain at a 1∶1 molar ratio was clearly observed. This differential line broadening originates from the affected nuclei at the binding interface of the proteins upon complex formation and results in a significant decrease in the intensity of a subset of amide cross peaks in the ^1^H-^15^N HSQC spectrum. Previous NMR studies on S100 proteins with their target proteins have also reported that the residues most affected by complex formation will experience substantial changes in the NMR resonance line width and forms the basis for the identification of the interfacial features of the protein-protein complex [22], [73]. The current study presents a similar scenario. Thus, to identify the S100P residues that interact with the RAGE V domain, changes in the HSQC cross-peak intensities of the S100P-RAGE V domain complex (I) in comparison to free S100P (I_0_) were plotted in a bar graph, as shown in **Figure 3b**. Analysis of the bar graph indicates that most of the residues that exhibited decreases in cross-peak intensity clustered in helix-1′ (residues 2–14), the linker region (residues 43–49), and helix-4 (residues 88–93), forming one continuous surface on both monomers of the S100P homodimer, as shown in **Figure 3c**. Differential line broadening of NMR resonances was also observed in the reciprocal HSQC titration experiments using ^15^N-labeled RAGE V domain with unlabeled S100P at a 1∶1 molar ratio. These experiments were performed to identify the RAGE V domain residues that interact with S100P. A subset of cross peaks that exhibited altered intensities due to affected nuclear signals of RAGE V domain residues upon S100P binding, such as R48, K52, N54, W61, K62, Q67, G68, L86, C99, Q100, R104, N105 and K110, are shown in **Figure 4a**. A bar graph of the change in the HSQC cross-peak intensities of the RAGE V domain-S100P complex (I) compared with the free RAGE V domain (I_0_) was used to map the critical residues involved in complex formation and is shown in **Figure 4b**. The residues that exhibited a large decrease in cross-peak intensities were mapped onto the RAGE V domain structure to define the S100P binding site and distributed over discontinuous regions comprising of L4, L6 and L8 loops and the β3 and β6 strands, as shown in **Figure 4c**. Previous studies have described the importance of the interfacial residues R48, K52, R104 and K110 in the interaction of the RAGE V domain with its known binding partners, including S100B, S100A6 and AGE [22], [44], [74], [75]. The role of these solvent accessible residues has been further substantiated in this analysis of the S100P-RAGE V domain complex. Furthermore, to characterize the exchange regime of binding between free S100P and S100P in complex with the RAGE V domain, HSQC analysis using ^15^N-labeled S100P and unlabeled RAGE V domain were performed at higher molar ratios (1∶2, 1∶3, 1∶4) to monitor the rate of association-dissociation equilibrium between free and complexed S100P. During the high molar ratio titrations, a majority of the resonances exhibited significant line broadening (data not shown). Overall, the observed spectral changes reflect the intermediate exchange regime between free S100P and S100P in complex with the RAGE V domain.

### 3.4 Structural model of the S100P-RAGE V domain complex

**Figure 5:**
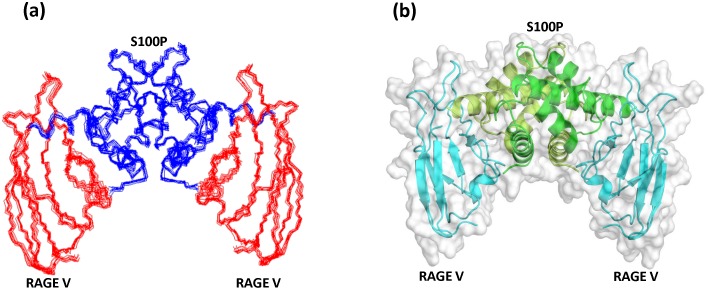
Model of the S100P-RAGE V domain complex determined by HADDOCK. (a) Backbone structures of an ensemble of the 10 lowest energy structures of the S100P-RAGE V domain complex. The S100P and RAGE V domain backbones are colored dark blue and red, respectively. (b) Ribbon and surface representation of the S100P-RAGE V domain complex, with the secondary structure elements of each RAGE V domain colored cyan and the secondary structure elements of the S100P homodimer colored green and lemon green.

After mapping the binding interface using NMR HSQC titration experiments, we sought to model the structure of the S100P-RAGE V domain complex to characterize the molecular interactions. Ambiguous interaction restraints were obtained from the changes in the resonance line width and shape of the affected residues in S100P and the RAGE V domain from the ^15^N-HSQC titration experiments (**Table S1 in File S1**). Most of these residues are surface exposed in the free proteins and form one continuous interface in the protein-protein complex, indicating localized changes in the spatial geometries of both S100P and the RAGE V domain. These findings thus support the NMR restraint-driven HADDOCK approach utilized to generate the structure of the heterotetrameric complex of S100P and the RAGE V domain [76], [77]. The structural coordinates for calcium-bound S100P and the NMR solution structure of the V domain of RAGE were obtained from the Protein Data Bank (PDB). The intermolecular nuclear Overhauser effect (NOE) is an phenomenon that allows the unambiguous mapping of bimolecular interactions. Intermolecular NOEs are invaluable in resolving the ambiguities that arise due to different possible orientations between protein partners during the docking process. Ten intermolecular NOEs were identified from ^13^C-filtered experiments and were used as unambiguous distance restraints in docking (**Figure S1 in File S1**). A set of 2000 complex structures was generated by rigid-body minimization and was further refined to the best 200 structures based on the total energy, selecting for torsion angle dynamics and subsequent Cartesian dynamics in an explicit solvent (water) model to flexibly optimize the contacts. The 200 structures with the lowest water-refined interaction energies were used for subsequent analysis. HADDOCK clustered these 200 structures into a single cluster, with an RMSD of 0.6±0.3 from the overall lowest energy structure (**Figure S2 in File S1**). **Figure 5a** shows a stereo view of the backbone structures of the final S100P-RAGE V domain ensemble using the 10 lowest energy structures from cluster 1 derived from the HADDOCK calculations. **Figure 5b** shows the energy-minimized mean structure of the heterotetrameric S100P-RAGE V domain complex in ribbon and surface representation. Subsequent analysis indicated that the unambiguous NOEs are consistent with the calculated protein complex structure (**Figure S3 in File S1**). PROCHECK analysis of the structure indicated good stereochemistry for the bond lengths and bond angles; 99.4% of all of the non-glycine residues fall within the allowed region of the Ramachandran plot (**Table 1**). The structural coordinates have been deposited in the Protein Data Bank (PDB ID: 2MJW).

**Table 1:** HADDOCK structure calculation statistics of the 10 best S100P–RAGEV model structures.

Parameter	Value
**NMR restraints**	
Distance restraints	
Total NOE restraints	49
Total unambiguous NOE restraints	10
Ambiguous interaction restraints (AIR)	39
**CNS energies [kcal/mol] after water refinement**	
E_tot_	–877.3±73.3
E_vdw_	–154.5±13.9
E_electr_	–722.7±72.0
**Violation/quality analysis (dihedral violations)**	
Violations >5°	0.0±0.0
Violations >10°	0.0±0.0
**RMSD from idealized geometry**	
Bond (Å)	0.0054±0.0002
Angle (°)	0.69±0.02
**RMSD from average structure: S100P+RAGE V**	
Backbone	0.61
Heavy atoms	0.67
**PROCHECK analysis**	
Residues in most favored regions (%)	84.0
Residues in additional allowed regions (%)	15.3
Residues in generously allowed regions (%)	0.4
Residues in disallowed regions (%)	0.3

### 3.5 Binding interface of the S100P-RAGE V domain Complex

**Figure 6:**
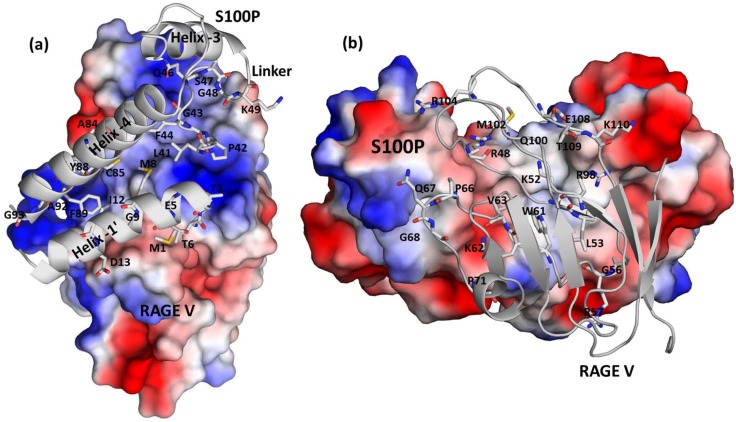
The interface of the S100P-RAGE V domain complex. (a) Electrostatic surface representation of the binding interface of the RAGE V domain in complex with S100P (gray ribbon), with the S100P residues involved in the interaction represented as sticks. (b) Electrostatic surface representation of S100P in complex with the RAGE V domain (gray ribbon), with the residues in the RAGE V domain that are involved in the interaction represented as sticks. Positively charged, negatively charged, and neutral regions are represented in blue, red and white, respectively. The atoms in S100P and the RAGE V domain are colored gray (carbon), red (oxygen) and blue (nitrogen).

The specificity of S100 proteins for their target proteins has been attributed to the variable nature of the binding surface, structural features and oligomerization states of individual S100 proteins [78]. A structural comparison of calcium-bound S100P with other S100 proteins indicated that the linker region of S100P is more flexible than that of other S100 proteins and plays a crucial role in the recognition of target proteins. Furthermore, Y88 and F89 in the C-terminal hydrophobic surface of S100P are known to play an important role in the recognition of target proteins, such as ezrin [26]. Calcium-bound S100P exposes its central linker region (residues 43–49), helix 4 (residues 83–94) of the first monomer and helix 1′ (residues 2–14) of the second monomer, creating a continuous binding surface for the RAGE V domain. The nonpolar residues of calcium-bound S100P, such as A92, G93, F89, Y88, G9, I12, M8, M1, F44, G43, P42, L41 and G48, create a hydrophobic patch that interacts with the RAGE V domain (**Figure 6a**). The acidic residues E5 and D13 of S100P prefer charge-charge interactions with the RAGE V domain, which potentially assist in the stabilization of the complex. Additional neutral and polar residues, including T6, T2, S47, Q46, and C85, provide nonhydrophobic interactions with the RAGE V domain. The identified RAGE V domain residues that interact with S100P primarily consist of basic residues, including R48, K52, K62, R98, R104 and K110, which form interspersed basic patches on one face of the RAGE V domain. Additional important residues, such as L53, W61, V63, P66, G68, G56, M102 and P71, around these basic patches form hydrophobic interactions with S100P **(** **Figure 6b** **)**. Moreover, the residues Q67, Q100, E108 and T109 also directly interact with S100P. Collectively, these residues form one continuous surface on the RAGE V domain.

### 3.6 Site-Directed Mutagenesis

**Figure 7:**
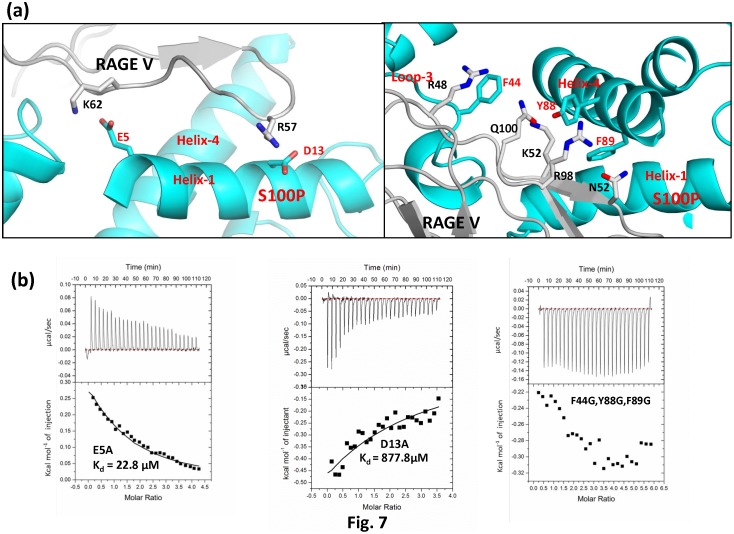
ITC titrations of the RAGE V domain with S100P mutants. (a) Ribbon representation of the model of the S100P-RAGE V domain complex with the mutated polar charged residues and hydrophobic residues of S100P shown as sticks. Helix-1, Helix-4 and loop-3 of S100P are colored cyan, and RAGE V is colored gray with the residues that interact with the mutated S100P residues shown as sticks. (b) Binding properties of S100P E5A, S100P D13A, and S100P F44G/Y88G/F89G with RAGE V domain. The upper panels represent the raw data and whereas the bottom panels are the integrated plot of the amount of heat liberated per injection as a function of the molar ratio of the S100P mutants to RAGE V domain. The dissociation constants (K_d_) for the RAGE V domain-S100P E5A interaction and the RAGE V domain-S100P D13A interaction were determined to be 23.6 µM and 877.8 µM, respectively. No binding of S100P F44G/Y88G/F89G to the RAGE V domain was detected.

Additional structural insights into the binding interface between calcium-bound S100P and the V domain of RAGE were obtained from ITC analysis of S100P mutants. Three mutants were designed to evaluate the contribution of the residues at the binding interface of the S100P-RAGE V domain complex. We mutated the S100P residues E5 and D13 to alanine to assess the role of crucial electrostatic interactions at the protein-protein interface. We also constructed an S100P triple mutant (F44G/Y88G/F89G) using site-directed mutagenesis of hydrophobic residues to glycine instead of the conventional alanine to completely abolish the hydrophobic side chain contributions of these residues at the binding interface, as shown in **Figure 7a**. The far-UV CD spectra of the wild-type and mutant S100P proteins were compared to characterize the effect of the mutations on the secondary structure of S100P. Analysis of the CD spectra indicated that the wild-type and mutant S100P proteins did not exhibit significant changes in the overall secondary structure of the protein (**Figure S4 in File S1**). Mutation of E5 to alanine produced a 3.8-fold reduction (22.8 µM) in the binding affinity and the S100P D13A mutant similarly exhibited a 146-fold reduction in the dissociation constant (877.8 µM) for the interaction with the RAGE V domain. However, no interaction was observed between the S100P F44G/Y88G/F89G triple mutant and the RAGE V domain, as shown in **Figure 7b** **.** The substantial reduction in the binding affinity of the S100P mutants in comparison to wild-type S100P suggests that E5, D13 and the aromatic residues act as ‘hotspot’ residues that are involved in crucial interactions with the RAGE V domain and, thus, are significant for protein-protein complex stability. An additional comparative analysis of the thermodynamic parameters of the E5A and D13A S100P mutants with wild-type S100P indicates modest variations in the enthalpic factor in contrast to significant deviations in the entropic factor (**Table S2 in File S1**). Complete abolition of binding between the S100P triple mutant and the RAGE V domain indicates the importance of hydrophobic residues in complex formation, i.e., mutations at the interface of the protein complex affect the buried surface area (BSA) between both proteins, confirming that the binding between S100P and the RAGE V domain was predominately driven by favorable entropic factors as proposed for the D13A and E5A mutants [79]. It is possible that the modulation of the binding interface by mutating the binding residues can predominantly alter either the binding entropy or the binding enthalpy during the course of the interaction, which was attributed to entropy/enthalpy compensation [80], [81].

### 3.7 Functional assay

**Figure 8:**
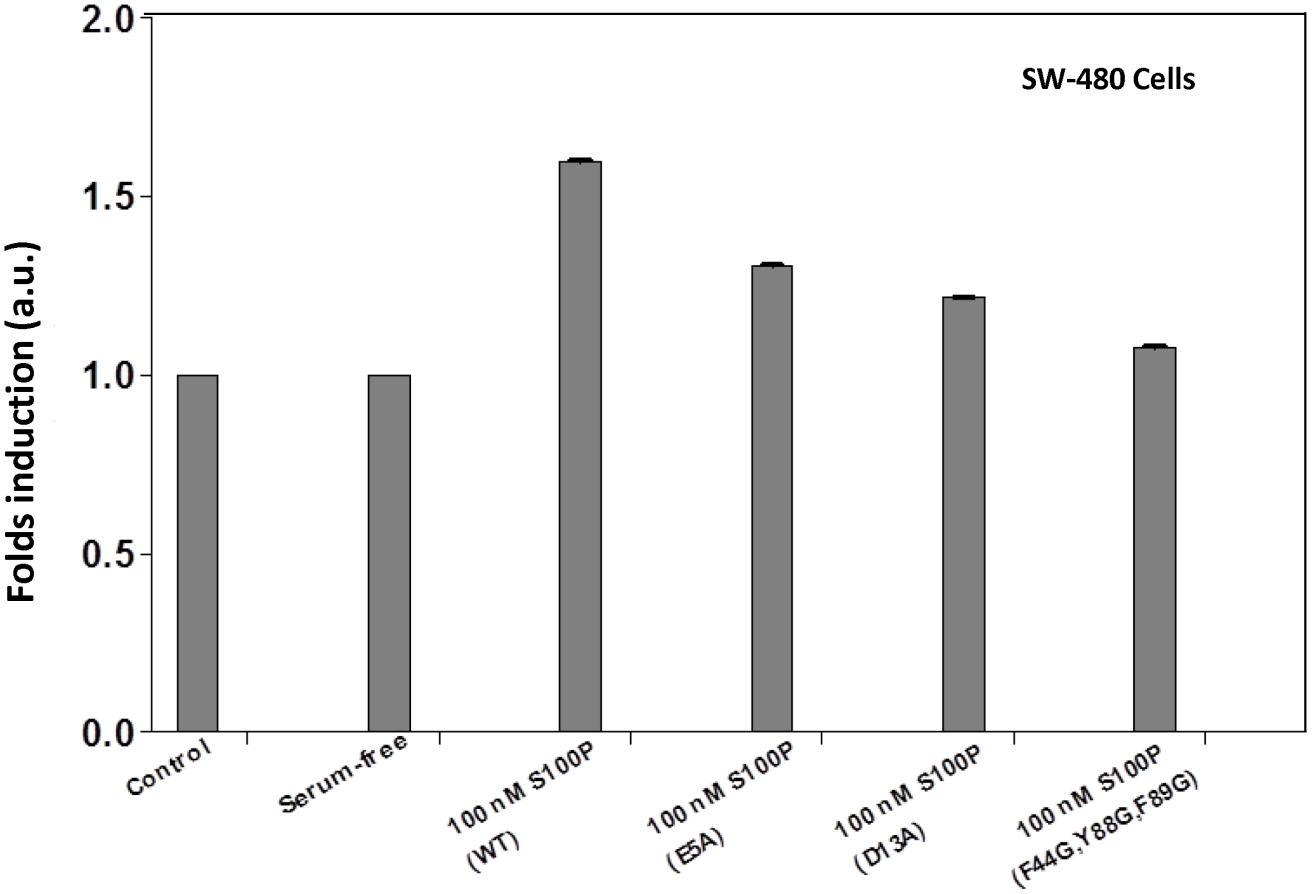
Functional assay. SW-480 cells were treated with 100 nM S100P, 100 nM S100P E5A, 100 nM S100P D13A, S100P F44G/Y88G/F89G or 10 µM FPS-ZM1, and cell proliferation was assessed using an MTT assay. The relative cell counts following treatment with the S100P mutants is plotted as the fold induction, with serum-free medium and FPS-ZM1 as the corresponding controls. The data are expressed as the means ± SD of 3 independent experiments.

S100P-mediated RAGE receptor activation has been demonstrated to be a critical step in the overall pathway for the subsequent downstream stimulation of cell proliferation in various cancer cell lines [29]. To determine the role of the critical S100P residues required for the interaction with the V domain of RAGE, the aforementioned site-directed mutants of S100P were used in a cell proliferation assay to further evaluate the overall biological changes produced during the interaction between these two proteins. SW-480 cells were chosen for this functional assay, and cells were treated with wild-type S100P and S100P mutants for 48 h. The relative number of viable cells was determined using an MTT assay. Serum-starved SW-480 cells were cultured followed by treatment with a nanomolar concentration of wild-type or mutant S100P. In the presence of 100 nM wild-type S100P, an approximately 1.5-fold increase in the viable cell count was observed compared with the control. However, upon treatment with the S100P mutants E5A or D13A, a significant reduction in the stimulation of cell proliferation was observed compared with wild-type S100P. In contrast, the triple mutant (F44G/Y88G/F89G) did not exhibit an increase in cell proliferation, as observed for cells treated with FPS-ZM1, which is a potent specific inhibitor that blocks ligand binding to the V domain of RAGE used in our study (**Figure 8**) [82]. The pronounced inhibitory effect leading to a significant reduction in cell proliferation following treatment with S100P mutants confirmed the biological significance of these residues at the interface of the S100P-RAGE V domain complex. The results from the functional assay are consistent with the reduced binding affinities between the S100P mutants and the RAGE V domain determined from ITC.

### 3.8 S100P-pentamidine interactions: a Small Molecule Inhibitor of the Calcium-Bound S100P-RAGE V Domain Complex

**Figure 9:**
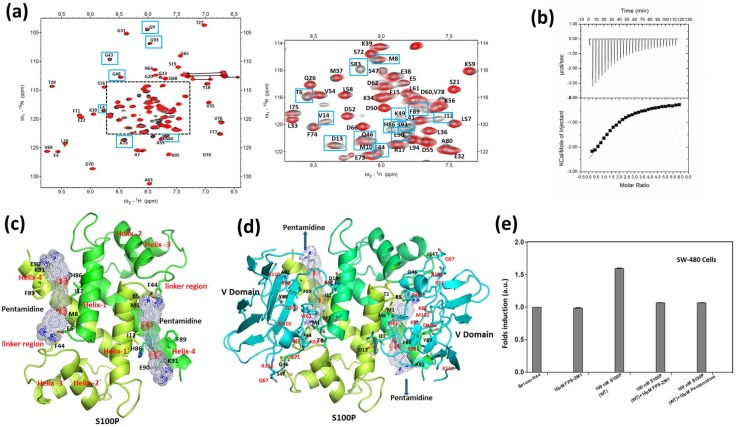
S100P-pentamidine interactions. (a) Overlaid ^1^H-^15^N HSQC spectra of uniformly ^15^N-labeled free S100P (black) and S100P upon binding pentamidine at a 1∶1 molar ratio (red). (b) Thermodynamic studies of S100P with pentamidine as a ligand. Raw data (upper panels) and integrated heat measurements (lower panels) as a function of the molar ratio of the pentamidine to S100P protein. The dissociation constant (K_d_) for this interaction is 305 µM at a 1∶1 stoichiometry. (c) Ribbon representation of the HADDOCK-modeled S100P-pentamidine complex. Monomers of the S100P homodimer are colored green and lemon green, with pentamidine shown as gray sticks. Structural elements of S100P are labeled in red. (d) Ribbon representation of the S100P-RAGE V domain complex with pentamidine at the binding interface. (e) Effects of pentamidine on S100P-mediated cell proliferation and RAGE signaling. SW-480 cells were treated with 10 µM FPS-ZM1, 100 µM S100P, 10 µM FPS-ZM1 plus 100 µM S100P or 10 µM pentamidine plus 100 µM S100P. Cell proliferation was analyzed after 48 h.

The binding of calcium-bound S100P to RAGE activates downstream signaling cascades in cancer cells. Blocking this interaction between S100P and RAGE has been considered an effective strategy to prevent various diseases. Previous reports suggest that various small molecules, such as cromolyn sodium, amlexanox, tranilast and pentamidine, bind to S100 family proteins [33], [83], [84], [85]. In this study, we used NMR-based screening approaches to characterize the interactions of these drugs with S100P and to identify the S100P binding interface for each of these drugs. Our NMR and ITC analysis demonstrated that pentamidine and cromolyn sodium bind to S100P, whereas amlexanox and tranilast do not bind to S100P (data not shown). In this study, the interactions of pentamidine with calcium-bound S100P were characterized using NMR spectroscopy, ITC, docking and cell proliferation studies. By comparing the calcium-bound S100P ^1^H-^15^N HSQC spectra in the absence and presence of pentamidine, we identified the S100P residues involved in pentamidine binding from the altered or missing cross-peaks in the calcium-bound S100P ^1^H-^15^N HSQC spectra, as shown in **Figure 9a**. Subsequent mapping studies revealed that these residues clustered in helix-1′ (L4, T6, G9, M10, I12, D13 and V14), the linker region (G43, F44, Q46, G48 and K49) and helix-4 (A79, A84, H86, F89, E90 and K91). ITC analysis of pentamidine binding to calcium-bound S100P resulted in a K_d_ value of 305 µM and indicated that the interaction was driven by enthalpy (ΔH = 7.2±0.12 kcal/mol) with a 1∶1 stoichiometry (**Figure 9b**). HADDOCK was again employed, using the interaction sites as constraints, to generate a potential model of the S100P-pentamidine complex (**Figure 9c**) using the X-ray crystal structures of calcium-bound S100P and pentamidine, the coordinates of which were obtained from the Protein Data Bank (PDB) and the Cambridge Structural Database (CSD) System, respectively. Our docking results suggest that pentamidine binds to a region in calcium-bound S100P that significantly overlaps with the binding site for the V domain of RAGE (**Figure 9d**). We further evaluated the cellular effects of pentamidine using a mitogenic assay and the SW-480 cell line. Here, we demonstrate that micromolar concentrations of pentamidine (10 µM) could effectively attenuate the biological functions of S100P, specifically its cell proliferation effect, which was comparable to the control FPS-ZM1 (**Figure 9e**). Wild-type S100P enhanced the cell proliferation by 1.5-fold. However, upon treatment with FPS-ZM1 and S100P, SW-480 cell proliferation was significantly reduced. Notably, FPS-ZM1 alone did not significantly affect cell proliferation. These results indicate that the cell proliferation activity of S100P is exclusive to S100P binding to the RAGE V domain. Our results suggest that pentamidine may represent a potential drug for the treatment of cancer by disrupting the interaction between S100P and the V domain of RAGE. The mechanistic studies defining the role of these antagonists for the S100P-RAGE complex would provide additional insight in understanding the pharmacological effects of these small molecules.

## Discussion

**Figure 10:**
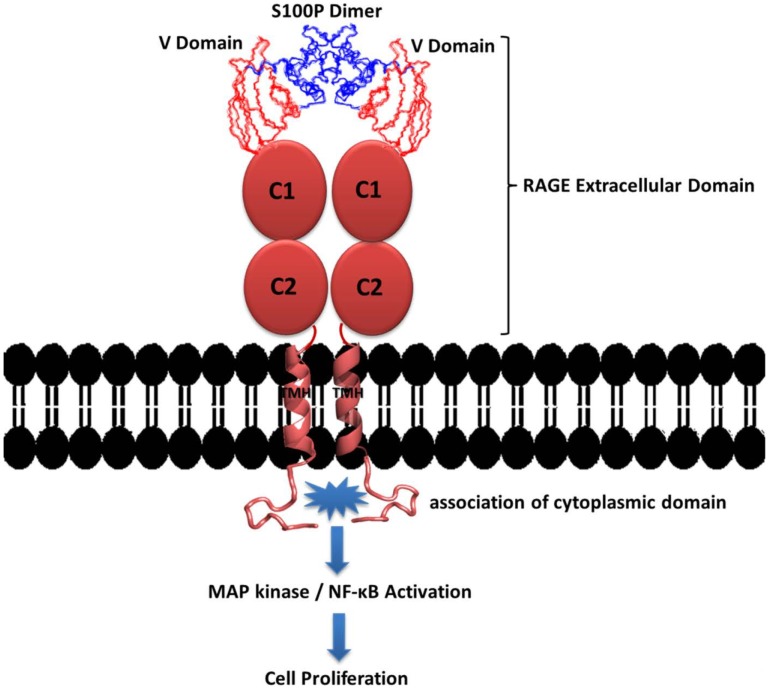
Proposed mechanism for the S100P-RAGE interaction. A proposed mechanism for the calcium-bound S100P-RAGE signaling cascade. Ligation of extracellular RAGE by S100P promotes the homodimerization of the cytoplasmic domain of RAGE and results in the activation of two pathways, MAPK and NF-κB, leading to the downstream activation of ERK1/2 (extracellular signal-regulated kinase) and subsequent cellular proliferation.

S100 proteins are calcium-binding proteins that represent a subfamily of EF-hand proteins. The members of this family share a common topology that consists of two calcium binding helix-loop-helix structural domains that mediate calcium-dependent signal transduction. These S100 proteins engage in a large number of intracellular and extracellular functions [86]. The binding of calcium to the EF-hand motif of S100 proteins induces conformational changes that facilitate the interaction of the hydrophobic interfaces on opposite sides of the homodimer with target proteins, thereby mediating their activity [42]. Previous studies strongly indicate that S100P can be secreted, acts through RAGE in an autocrine manner and plays a significant role in the development and progression of various cancers [87]. The binding of S100P to the RAGE V domain results in RAGE homodimerization and the activation of its cytoplasmic domain for autophosphorylation, leading to the activation of the ERK and MAPK pathways to mediate cell proliferation and survival (**Figure 10**). This finding indicates additional significance of the V domain in RAGE homodimerization and signal transduction. Therefore, the characterization of the molecular interactions between S100P and the V domain of RAGE provides an opportunity to precisely identify the role of crucial residues at the interface of both proteins. The interactions between the RAGE V domain and S100P were characterized using various biophysical techniques, including isothermal titration calorimetry (ITC), fluorescence spectroscopy, multidimensional NMR spectroscopy and site-directed mutagenesis. The dissociation constant (K_d_) estimated from ITC and fluorescence spectroscopy is approximately 6.0 µM, suggesting a moderately strong interaction. Our ITC results also suggested the presence of two identical and independent binding sites between two RAGE V domains and a calcium-bound S100P homodimer, supporting the hypothesis that hydrophobic interfaces are buried between the proteins upon complex formation. Additional NMR titration experiments identified the putative binding interface for the S100P-RAGE V domain complex. Mapping of the binding interface residues indicated the nature of the binding interfaces of S100P and the RAGE V domain. Overall, our findings reveal the extracellular role of the S100P homodimer, which symmetrically interacts with two RAGE V molecules. Based on these conclusions, we modeled the S100P-RAGE V heterotetrameric complex using HADDOCK, which revealed the putative interfaces between S100P and the RAGE V domain. An analysis of the total interface area for the HADDOCK-calculated S100P-RAGE V domain complex is approximately 4738 Å^2^ per two interfaces, with a ratio of the hydrophobic to hydrophilic areas of 58∶42. Interestingly, the RAGE V domain residues at the S100P binding interface, particularly R48, K52, R98 and R104, are similar to those that interact with S100B, S100A6 and AGE. Overall, we can infer that the binding interface of the RAGE V domain is well conserved for its interaction with S100P and is similar to that for other known RAGE V domain binding partners, as has been observed in the RAGE-AGE complex, the S100B-RAGE VC1 model complex, and the S100A6-RAGE V model complex [44]. Our docking results and mutagenesis study indicated that hydrophobic residues, such as F44 in the central linker region and Y88 and F89 in helix-4, and polar residues, including E5 and D13 in helix-1′, in S100P permitted the unique recognition of the RAGE V domain. Overall, our results demonstrated the ability of the S100P homodimer to form two symmetrical binding interfaces from helix 4 and the linker region of one monomer and helix 1′ from the other monomer to interact with RAGE. S100A12 was previously shown to bind to the C1C2 domain of RAGE in a similar fashion using two symmetrical hydrophobic patches from helix-2, loop-2 and helix-4 of one monomer and helix-1′ of a second monomer [88]. Furthermore, the differences in the S100P binding interface for RAGE V domain complex formation can be ascertained from the reported interactions between various S100 proteins and the RAGE V domain. It has been reported that the binding interface between S100B and the RAGE V domain consists of H42, V52, N62, D62, D69, F70 and A78, most of which are located on helix-4, helix-3, loop-1 and loop-3 [22]. For S100A11, the V domain-binding residues are located in helix-2 and helix-4 [89]. The model of the S100A6-RAGE V domain complex suggests that the RAGE V domain-binding surface of S100A6 is predominantly distributed over loop 1, loop 3 and helix 4 [75]. These differences in the binding interfaces between various S100 proteins and RAGE suggest that RAGE recognizes its S100 ligands based on their net charge, their polarity and the hydrophobic nature of the interface. The modeled structure of the S100P-RAGE V domain complex is useful for the improvement of current drug antagonists and may aid in the design of improved antagonists to disrupt the interaction between S100P and the V domain of RAGE. We identified pentamidine as a potential small molecule antagonist that disrupts this interaction using NMR and ITC analysis, HADDOCK modeling and mitogenic assays. Our present findings highlight the significance of cell proliferation induced by the interaction of S100P with RAGE. This study will also aid in the design of improved therapeutics to antagonize the S100P-RAGE interaction to prevent RAGE-dependent diseases.

## Supporting Information

File S1 **Combined file containing supporting figures and tables. Table S1.** Active and passive residues used to define the ambiguous interaction restraints for the docking of S100P with the V domain of RAGE. **Table S2.** Thermodynamic parameters of the interaction between wild-type or mutant S100P and the V domain of RAGE, as determined by ITC. K_d_, dissociation constant; ΔH and ΔS, changes in the enthalpy of binding and entropy of binding, respectively; ΔG_binding_, Gibbs free energy of binding; T, temperature in Kelvin; and ΔG_binding_ = ΔH − TΔS. **Figure S1.** Intermolecular NOEs between the V domain of RAGE* and S100P. The intermolecular NOE peaks between the V domain of RAGE and S100P were observed in ^13^C(ω2)-edited, ^12^C(ω3)-filtered NOESY-HSQC experiments and are represented as strip plots. **Figure S2.** Scatter plot of the HADDOCK score versus the fraction of native contacts (FCC) for a single cluster generated by HADDOCK. **Figure S3.** Detailed view of Intermolecular NOEs between residues in the RAGE V domain (green) and S100P (cyan) of the modeled RAGE V domain-S100P complex. **Figure S4.** Secondary structure characterization of wild-type S100P and S100P mutants. Each protein was measured at a concentration of 32 µM in 20 mM Tris-HCl (pH 7.0), 100 mM NaCl, and 4 mM CaCl_2_. An average of three far-UV CD spectra scans were recorded for each S100P protein from 195 nm to 260 nm using a JASCO-720 spectropolarimeter.
(ZIP)
