# Supplementary figures and images for: Structural Insights into Calcium-Bound S100P and the V Domain of the RAGE Complex
Source: PLoS One. 2014 Aug 1;9(8):e103947. doi: 10.1371/journal.pone.0103947 (PMC4118983; doi:10.1371/journal.pone.0103947)

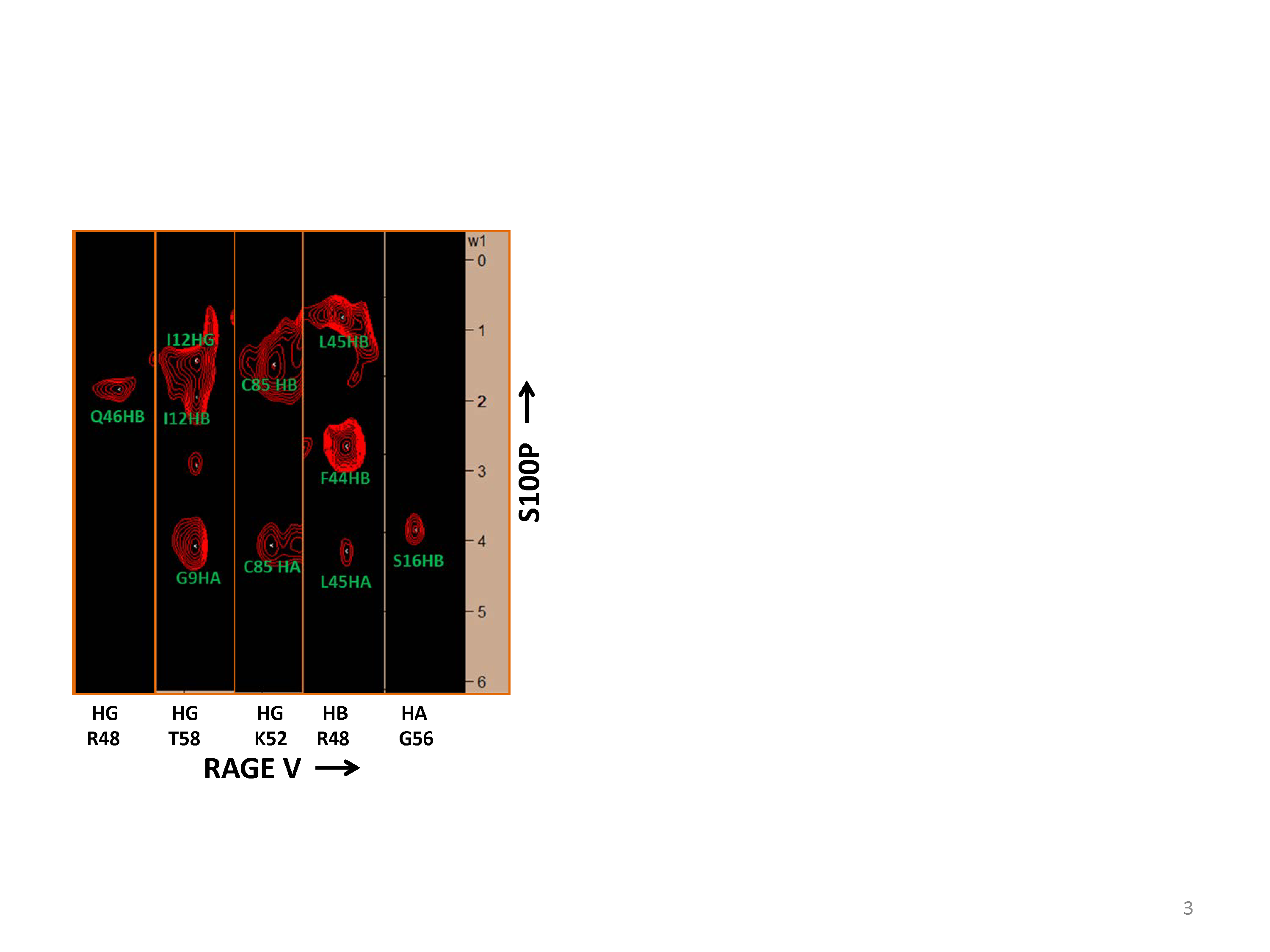

Supplement: File S1 — Combined file containing supporting figures and tables. Table S1. Active and passive residues used to define the ambiguous interaction restraints for the docking of S100P with the V domain of RAGE. Table S2. Thermodynamic parameters of the interaction between wild-type or mutant S100P and the V domain of RAGE, as determined by ITC. Kd, dissociation constant; ΔH and ΔS, changes in the enthalpy of binding and entropy of binding, respectively; ΔGbinding, Gibbs free energy of binding; T, temperature in Kelvin; and ΔGbinding = ΔH − TΔS. Figure S1. Intermolecular NOEs between the V domain of RAGE* and S100P. The intermolecular NOE peaks between the V domain of RAGE and S100P were observed in 13C(ω2)-edited, 12C(ω3)-filtered NOESY-HSQC experiments and are represented as strip plots. Figure S2. Scatter plot of the HADDOCK score versus the fraction of native contacts (FCC) for a single cluster generated by HADDOCK. Figure S3. Detailed view of Intermolecular NOEs between residues in the RAGE V domain (green) and S100P (cyan) of the modeled RAGE V domain-S100P complex. Figure S4. Secondary structure characterization of wild-type S100P and S100P mutants. Each protein was measured at a concentration of 32 µM in 20 mM Tris-HCl (pH 7.0), 100 mM NaCl, and 4 mM CaCl2. An average of three far-UV CD spectra scans were recorded for each S100P protein from 195 nm to 260 nm using a JASCO-720 spectropolarimeter. (ZIP) [file pone.0103947.s001.zip › supp info plos/Figure S1.tiff]

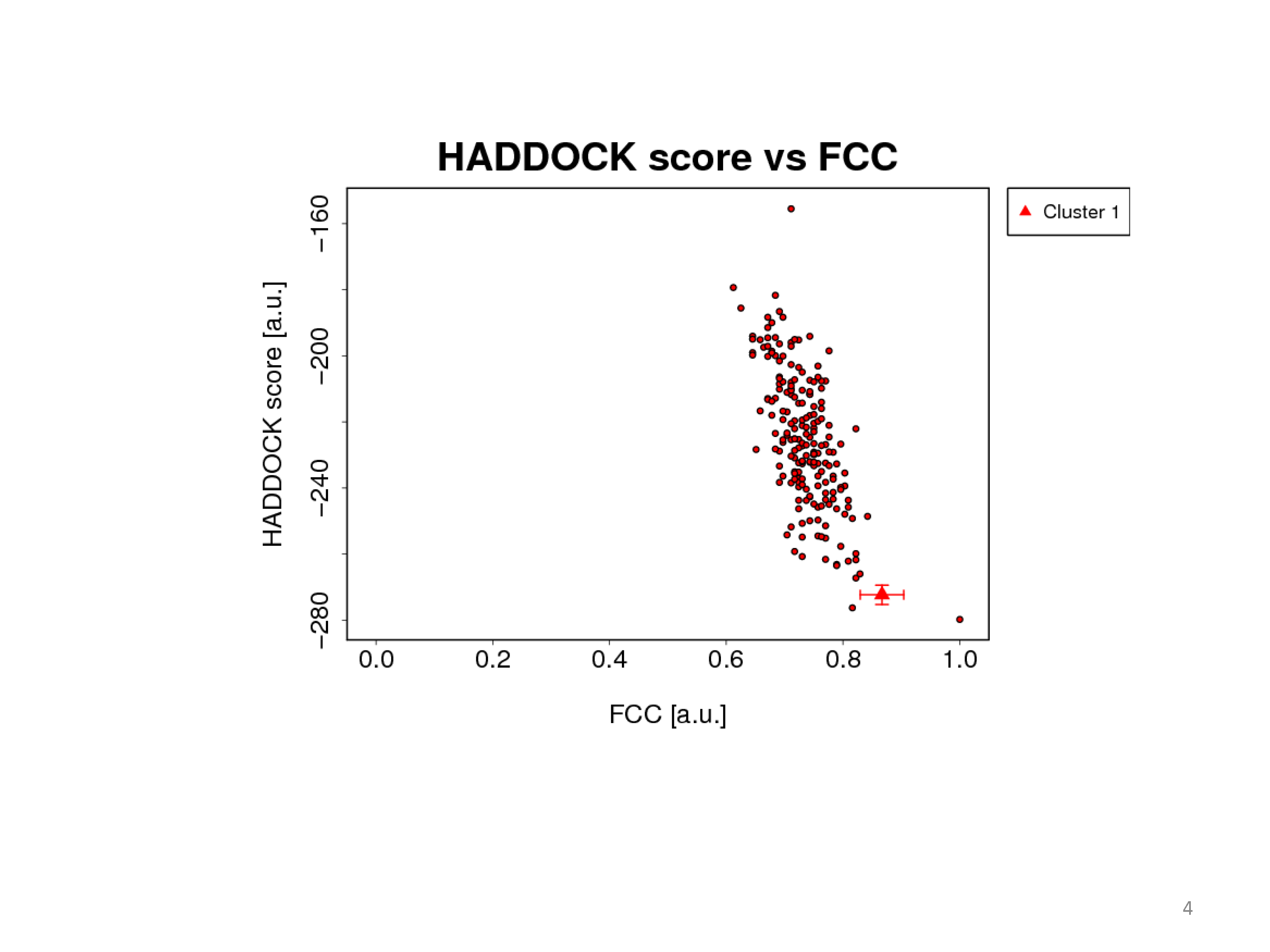

Supplement: File S1 — Combined file containing supporting figures and tables. Table S1. Active and passive residues used to define the ambiguous interaction restraints for the docking of S100P with the V domain of RAGE. Table S2. Thermodynamic parameters of the interaction between wild-type or mutant S100P and the V domain of RAGE, as determined by ITC. Kd, dissociation constant; ΔH and ΔS, changes in the enthalpy of binding and entropy of binding, respectively; ΔGbinding, Gibbs free energy of binding; T, temperature in Kelvin; and ΔGbinding = ΔH − TΔS. Figure S1. Intermolecular NOEs between the V domain of RAGE* and S100P. The intermolecular NOE peaks between the V domain of RAGE and S100P were observed in 13C(ω2)-edited, 12C(ω3)-filtered NOESY-HSQC experiments and are represented as strip plots. Figure S2. Scatter plot of the HADDOCK score versus the fraction of native contacts (FCC) for a single cluster generated by HADDOCK. Figure S3. Detailed view of Intermolecular NOEs between residues in the RAGE V domain (green) and S100P (cyan) of the modeled RAGE V domain-S100P complex. Figure S4. Secondary structure characterization of wild-type S100P and S100P mutants. Each protein was measured at a concentration of 32 µM in 20 mM Tris-HCl (pH 7.0), 100 mM NaCl, and 4 mM CaCl2. An average of three far-UV CD spectra scans were recorded for each S100P protein from 195 nm to 260 nm using a JASCO-720 spectropolarimeter. (ZIP) [file pone.0103947.s001.zip › supp info plos/Figure S2.tiff]

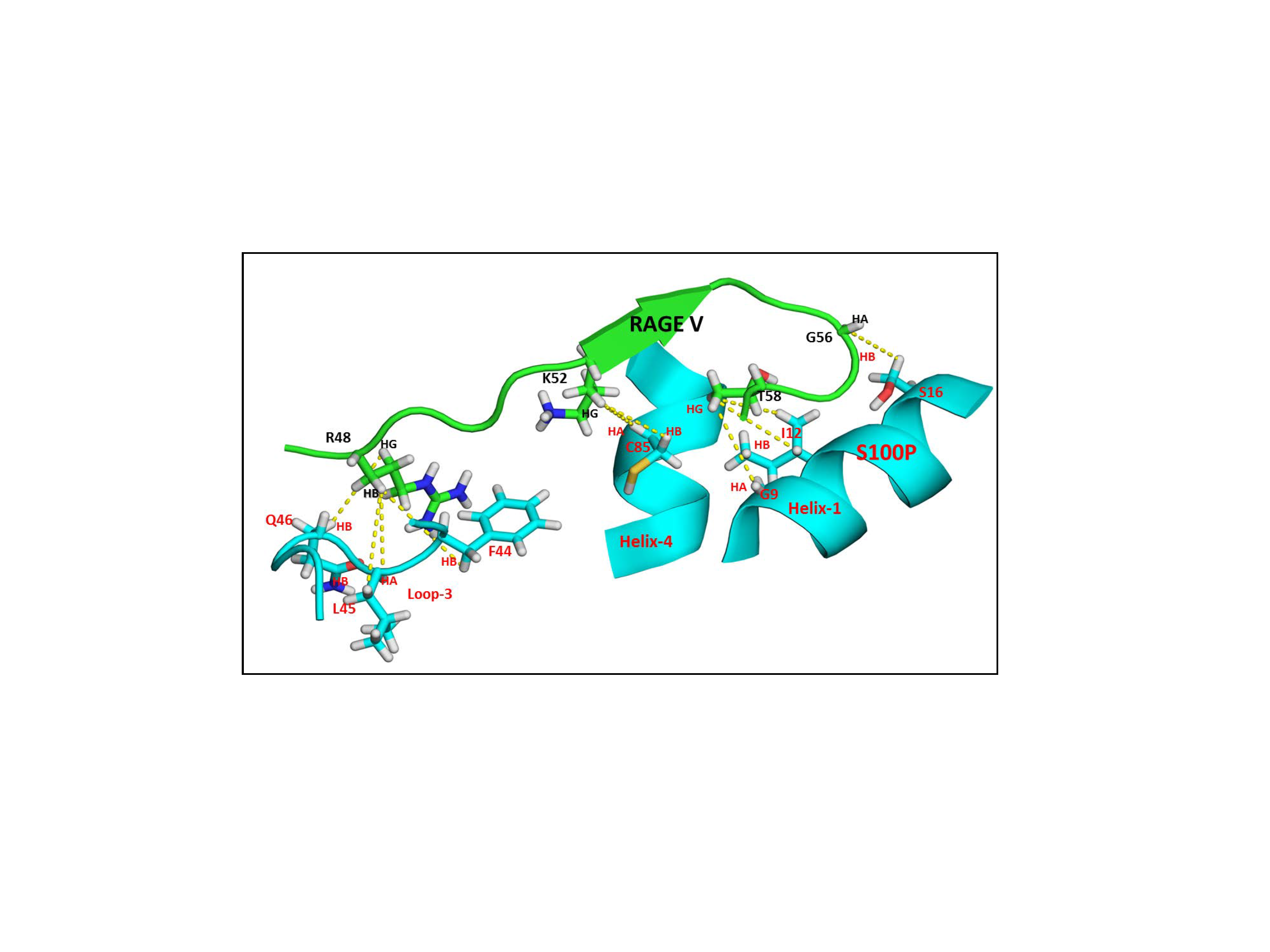

Supplement: File S1 — Combined file containing supporting figures and tables. Table S1. Active and passive residues used to define the ambiguous interaction restraints for the docking of S100P with the V domain of RAGE. Table S2. Thermodynamic parameters of the interaction between wild-type or mutant S100P and the V domain of RAGE, as determined by ITC. Kd, dissociation constant; ΔH and ΔS, changes in the enthalpy of binding and entropy of binding, respectively; ΔGbinding, Gibbs free energy of binding; T, temperature in Kelvin; and ΔGbinding = ΔH − TΔS. Figure S1. Intermolecular NOEs between the V domain of RAGE* and S100P. The intermolecular NOE peaks between the V domain of RAGE and S100P were observed in 13C(ω2)-edited, 12C(ω3)-filtered NOESY-HSQC experiments and are represented as strip plots. Figure S2. Scatter plot of the HADDOCK score versus the fraction of native contacts (FCC) for a single cluster generated by HADDOCK. Figure S3. Detailed view of Intermolecular NOEs between residues in the RAGE V domain (green) and S100P (cyan) of the modeled RAGE V domain-S100P complex. Figure S4. Secondary structure characterization of wild-type S100P and S100P mutants. Each protein was measured at a concentration of 32 µM in 20 mM Tris-HCl (pH 7.0), 100 mM NaCl, and 4 mM CaCl2. An average of three far-UV CD spectra scans were recorded for each S100P protein from 195 nm to 260 nm using a JASCO-720 spectropolarimeter. (ZIP) [file pone.0103947.s001.zip › supp info plos/Figure S3.tiff]

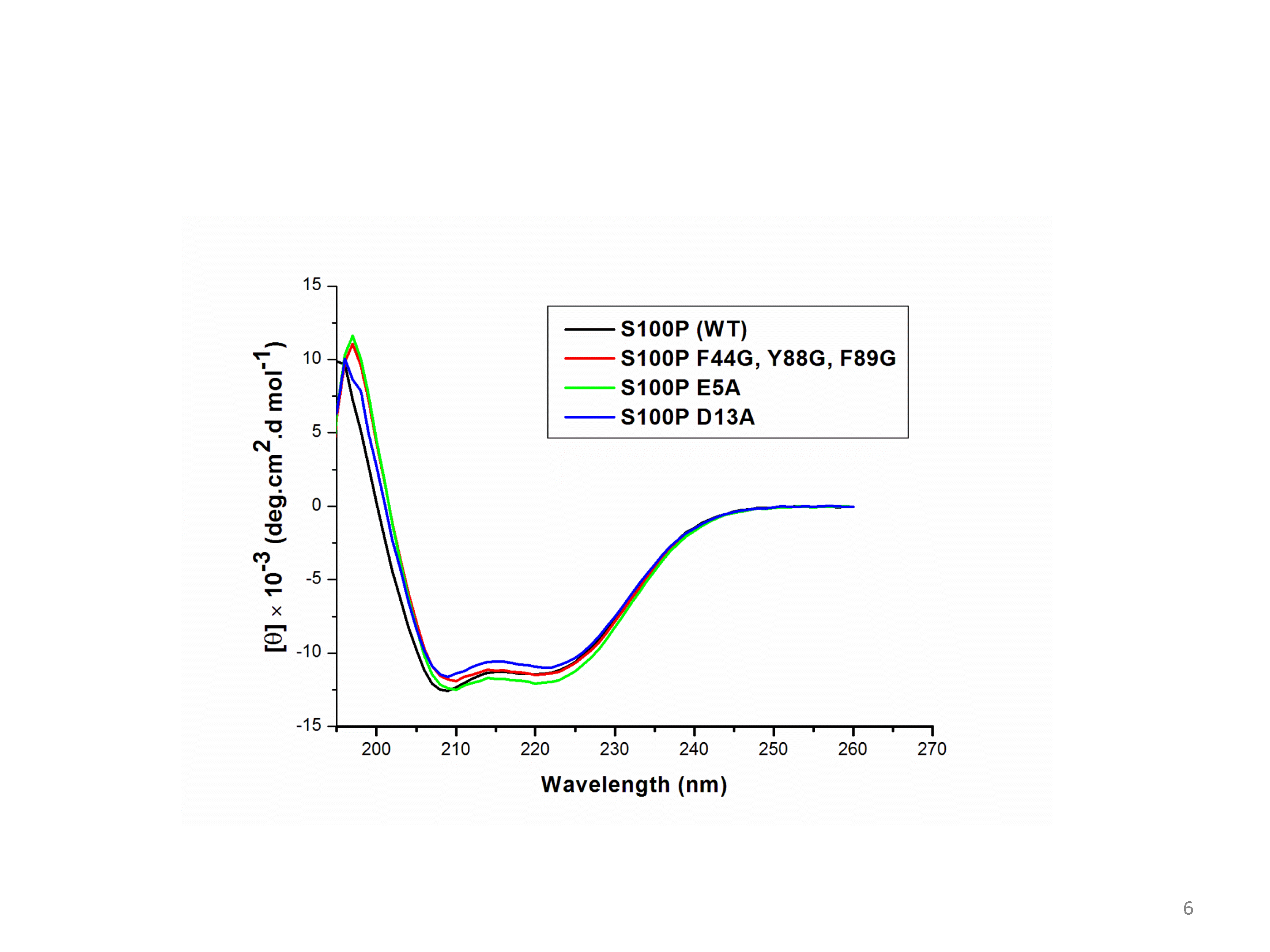

Supplement: File S1 — Combined file containing supporting figures and tables. Table S1. Active and passive residues used to define the ambiguous interaction restraints for the docking of S100P with the V domain of RAGE. Table S2. Thermodynamic parameters of the interaction between wild-type or mutant S100P and the V domain of RAGE, as determined by ITC. Kd, dissociation constant; ΔH and ΔS, changes in the enthalpy of binding and entropy of binding, respectively; ΔGbinding, Gibbs free energy of binding; T, temperature in Kelvin; and ΔGbinding = ΔH − TΔS. Figure S1. Intermolecular NOEs between the V domain of RAGE* and S100P. The intermolecular NOE peaks between the V domain of RAGE and S100P were observed in 13C(ω2)-edited, 12C(ω3)-filtered NOESY-HSQC experiments and are represented as strip plots. Figure S2. Scatter plot of the HADDOCK score versus the fraction of native contacts (FCC) for a single cluster generated by HADDOCK. Figure S3. Detailed view of Intermolecular NOEs between residues in the RAGE V domain (green) and S100P (cyan) of the modeled RAGE V domain-S100P complex. Figure S4. Secondary structure characterization of wild-type S100P and S100P mutants. Each protein was measured at a concentration of 32 µM in 20 mM Tris-HCl (pH 7.0), 100 mM NaCl, and 4 mM CaCl2. An average of three far-UV CD spectra scans were recorded for each S100P protein from 195 nm to 260 nm using a JASCO-720 spectropolarimeter. (ZIP) [file pone.0103947.s001.zip › supp info plos/Figure S4.tiff]

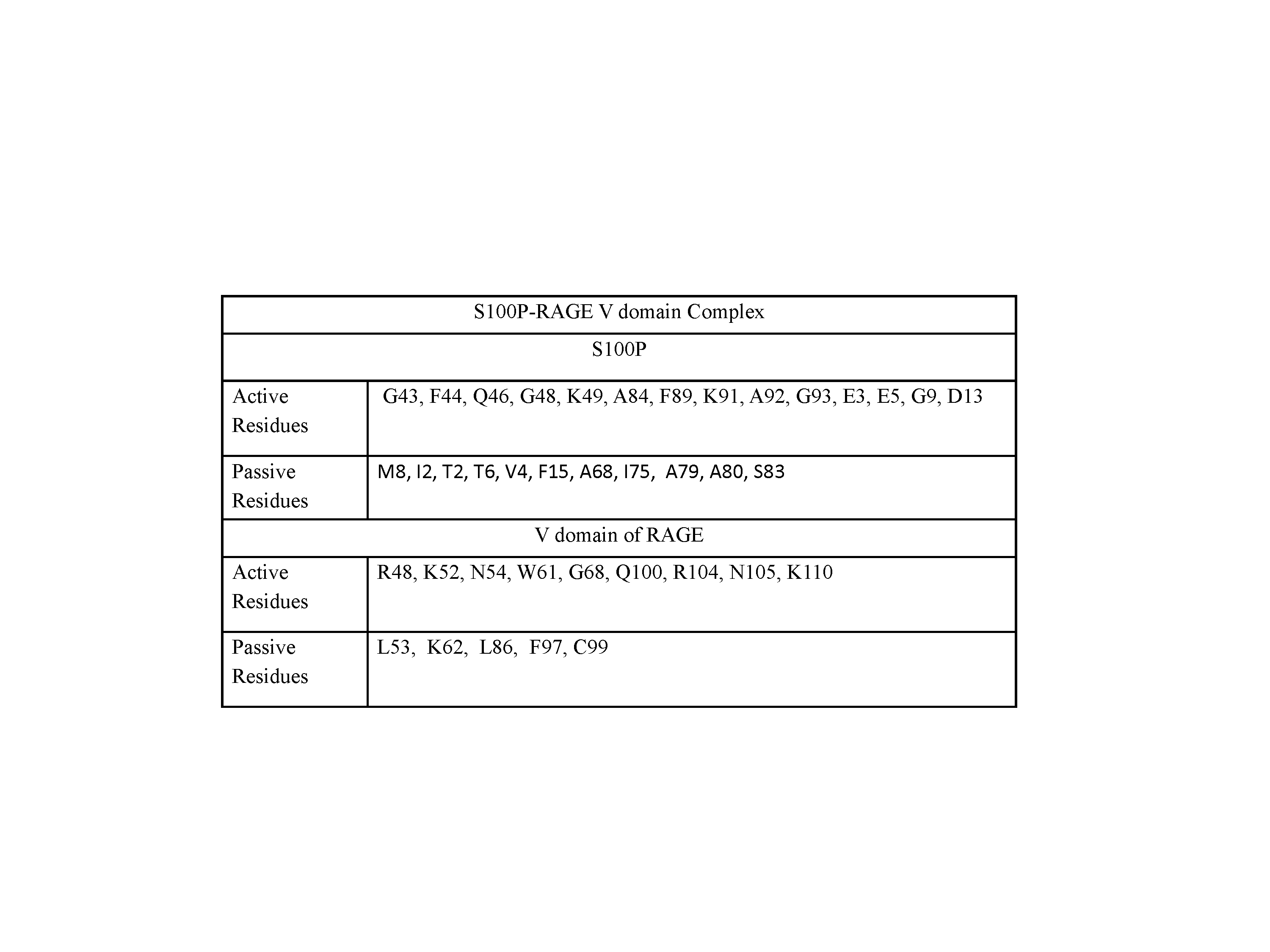

Supplement: File S1 — Combined file containing supporting figures and tables. Table S1. Active and passive residues used to define the ambiguous interaction restraints for the docking of S100P with the V domain of RAGE. Table S2. Thermodynamic parameters of the interaction between wild-type or mutant S100P and the V domain of RAGE, as determined by ITC. Kd, dissociation constant; ΔH and ΔS, changes in the enthalpy of binding and entropy of binding, respectively; ΔGbinding, Gibbs free energy of binding; T, temperature in Kelvin; and ΔGbinding = ΔH − TΔS. Figure S1. Intermolecular NOEs between the V domain of RAGE* and S100P. The intermolecular NOE peaks between the V domain of RAGE and S100P were observed in 13C(ω2)-edited, 12C(ω3)-filtered NOESY-HSQC experiments and are represented as strip plots. Figure S2. Scatter plot of the HADDOCK score versus the fraction of native contacts (FCC) for a single cluster generated by HADDOCK. Figure S3. Detailed view of Intermolecular NOEs between residues in the RAGE V domain (green) and S100P (cyan) of the modeled RAGE V domain-S100P complex. Figure S4. Secondary structure characterization of wild-type S100P and S100P mutants. Each protein was measured at a concentration of 32 µM in 20 mM Tris-HCl (pH 7.0), 100 mM NaCl, and 4 mM CaCl2. An average of three far-UV CD spectra scans were recorded for each S100P protein from 195 nm to 260 nm using a JASCO-720 spectropolarimeter. (ZIP) [file pone.0103947.s001.zip › supp info plos/Table S1.tiff]

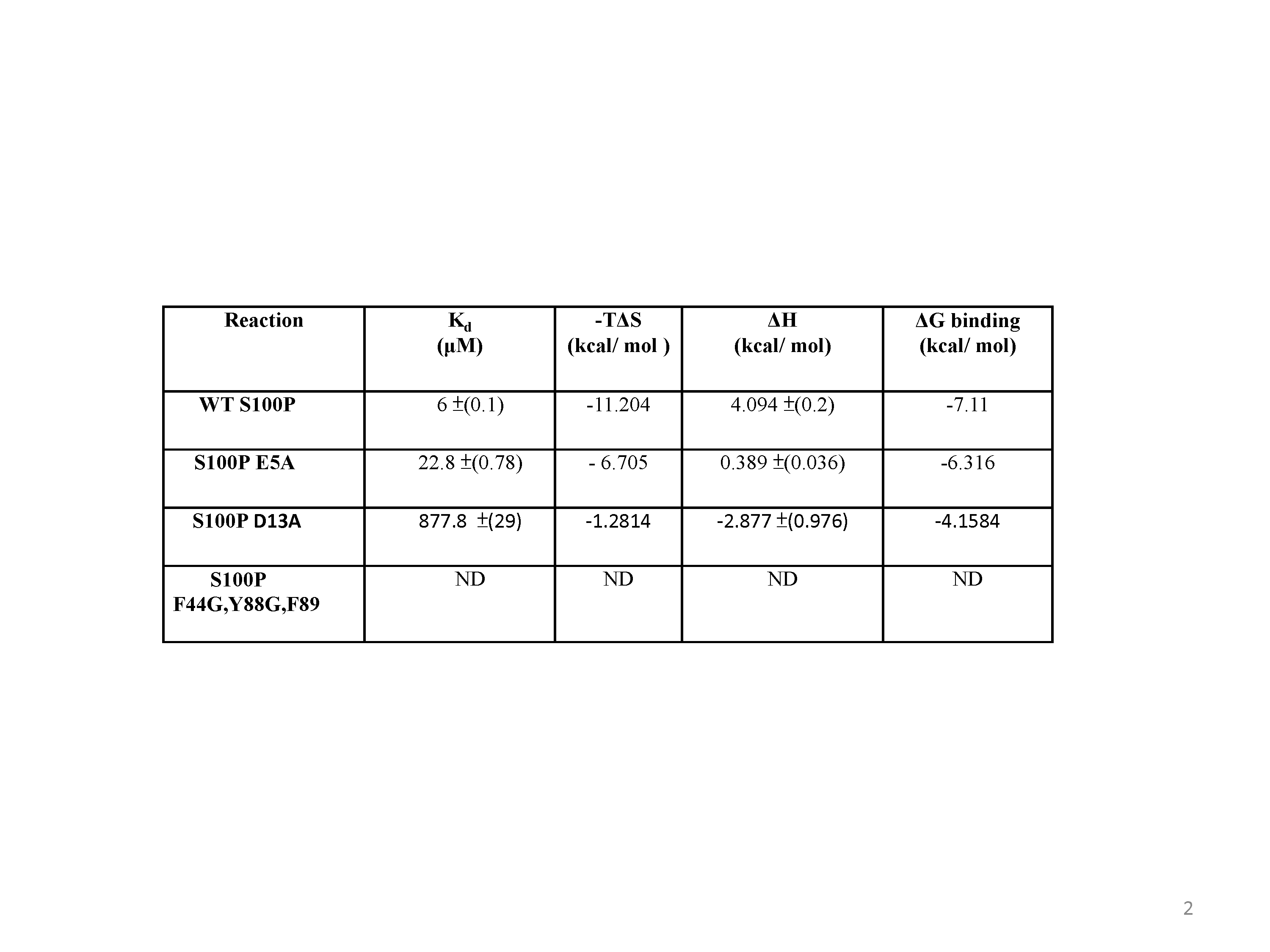

Supplement: File S1 — Combined file containing supporting figures and tables. Table S1. Active and passive residues used to define the ambiguous interaction restraints for the docking of S100P with the V domain of RAGE. Table S2. Thermodynamic parameters of the interaction between wild-type or mutant S100P and the V domain of RAGE, as determined by ITC. Kd, dissociation constant; ΔH and ΔS, changes in the enthalpy of binding and entropy of binding, respectively; ΔGbinding, Gibbs free energy of binding; T, temperature in Kelvin; and ΔGbinding = ΔH − TΔS. Figure S1. Intermolecular NOEs between the V domain of RAGE* and S100P. The intermolecular NOE peaks between the V domain of RAGE and S100P were observed in 13C(ω2)-edited, 12C(ω3)-filtered NOESY-HSQC experiments and are represented as strip plots. Figure S2. Scatter plot of the HADDOCK score versus the fraction of native contacts (FCC) for a single cluster generated by HADDOCK. Figure S3. Detailed view of Intermolecular NOEs between residues in the RAGE V domain (green) and S100P (cyan) of the modeled RAGE V domain-S100P complex. Figure S4. Secondary structure characterization of wild-type S100P and S100P mutants. Each protein was measured at a concentration of 32 µM in 20 mM Tris-HCl (pH 7.0), 100 mM NaCl, and 4 mM CaCl2. An average of three far-UV CD spectra scans were recorded for each S100P protein from 195 nm to 260 nm using a JASCO-720 spectropolarimeter. (ZIP) [file pone.0103947.s001.zip › supp info plos/Table S2.tiff]
